# Echolocation may have real-life advantages for blind people: an analysis of survey data

**DOI:** 10.3389/fphys.2013.00098

**Published:** 2013-05-08

**Authors:** Lore Thaler

**Affiliations:** Department of Psychology, Durham UniversityDurham, UK

**Keywords:** vision loss, blindness, adaptation, mobility, correlation, regression

## Abstract

Some people can echolocate by making sonar emissions (e.g., mouth-clicks, finger snaps, feet shuffling, humming, cane tapping, etc.) and listening to the returning echoes. To date there are no statistics available about how many blind people use echolocation, but anecdotal reports in the literature suggest that perhaps between 20 and 30% of totally blind people may use it, suggesting that echolocation affords broad functional benefits. Consistent with the notion that blind individuals benefit from the use of echolocation, previous research conducted under controlled experimental conditions has shown that echolocation improves blind people's spatial sensing ability. The current study investigated if there is also evidence for functional benefits of echolocation in real life. To address this question the current study conducted an online survey. Thirty-seven blind people participated. Linear regression analyses of survey data revealed that, while statistically controlling for participants' gender, age, level of visual function, general health, employment status, level of education, Braille skill, and use of other mobility means, people who use echolocation have higher salary, and higher mobility in unfamiliar places, than people who do not use echolocation. The majority of our participants (34 out of 37) use the long cane, and all participants who reported to echolocate, also reported to use the long cane. This suggests that the benefit of echolocation that we found might be conditional upon the long cane being used as well. The investigation was correlational in nature, and thus cannot be used to determine causality. In addition, the sample was small (*N* = 37), and one should be cautious when generalizing the current results to the population. The data, however, are consistent with the idea that echolocation offers real-life advantages for blind people, and that echolocation may be involved in peoples' successful adaptation to vision loss.

## Introduction

Some people, just like certain echolocating bats and marine mammals, can echolocate by making sonar emissions (e.g., mouth-clicks, finger snaps, feet shuffling, humming, cane tapping, etc.) and listening to the returning echoes (Stoffregen and Pittenger, 1995; Schenkman and Nilsson, 2010; Teng and Whitney, 2011). Echolocation can be learned by both blind and sighted people with normal hearing (Worchel and Mauney, 1951; Ammons et al., 1953; Teng and Whitney, 2011). Blind people are typically better at echolocation than sighted people, yet some sighted people can approach the accuracy of blind echolocation experts (Teng and Whitney, 2011). Recent research suggests that even the sighted human brain has cortical areas devoted to the processing of echoes (Thaler et al., under review). It is possible that the blind human brain capitalizes on these “pre-mordial” echolocation areas when acquiring echolocation skills (Thaler et al., 2011). To date there are no statistics available about how many blind people use echolocation, but anecdotal reports in the literature suggest that perhaps between 20 and 30% of totally blind people may do so (Wölfflin, 1909; Lamarque, 1929; Villey-Desmeserets, 1930).

The question arises what functional benefits people experience through the use of echolocation. In the context of bats it has been suggested that echolocation skills may have been naturally selected for, because they offer functional advantages, such as improved spatial orientation and/or acquisition of food (e.g., Schnitzler et al., 2003). Following this line of reasoning, we might hypothesize that blind people echolocate, because it offers broad functional benefits for them as well. Echolocation abilities in certain bats are the result of millions of years of evolution (Neuweiler, 2003; Denzinger et al., 2004). In contrast, a person's visual impairment and echolocation ability arise during that person's lifespan. I want to emphasize, therefore that here I am using the comparison to bats to emphasize potential analogies in terms of functional benefits, not to emphasize potential analogies in terms of evolutionary mechanisms. From the hypothesis that blind people use echolocation, because it offers functional benefits follows that there should be measurable functional benefits for blind people who echolocate, as compared to blind people who do not echolocate. Consistent with the hypothesis that echolocation offers functional benefits, previous research conducted under controlled experimental conditions has shown that echolocation improves blind people's spatial sensing ability, in that it improves their ability to determine distance, location, motion, size, shape or material of surfaces (for reviews see for example Stoffregen and Pittenger, 1995; Schenkman and Nilsson, 2010). Blind people can use echolocation for example to determine if a distant object is made out of denim, wood or metal (Kellogg, 1962), if a distant object is concave or flat (Thaler et al., 2011), to detect if there is a gap as small as 0.02 m between two objects placed 1 m away (Teng et al., 2012), to distinguish moving from stationary surfaces (Thaler et al., 2011) or to determine when a collision with an approaching wall is imminent (e.g., Supa et al., 1944; Cotzin and Dallenbach, 1950). It is an open question, however, to what degree functional improvements measured under controlled experimental conditions translate into benefits in real life [see for example Lane et al. (2008) for a discussion of this issue in the context of rehabilitative interventions]. Thus, the current study investigated if there is also evidence for functional benefits of echolocation in real life.

To address this question, the current study conducted a survey that was available on the internet and that was directed at blind people. Thirty-seven people participated in the survey. The survey solicited demographic information from participants, information about vision loss, general health and mobility. As indicators of participants' functional abilities I analysed data about participants' mobility, salary and relationship status. Mobility was defined by Long (1990) as “the ability to move about in the home and community” and by Foulke (1971) as “the ability to travel safely, comfortably, gracefully and independently through the environment.” Mobility was used as indicator for blind people's functional abilities, because vision loss has a negative impact on mobility (Brabyn, 1982; Brown and Brabyn, 1987; Long, 1990; Long et al., 1990; Deiaune, 1992; Salive et al., 1994; Roentgen et al., 2009). Based on this previous research, we also decided to assess mobility separately with regard to familiar and unfamiliar environments. Furthermore, because vision loss can be associated with a negative effect on salary (Tielsch et al., 1990, 1991; Houtenville, 2003) and the formation of romantic relationships (Van Hasselt, 1983; Huurre and Aro, 1998), these were also chosen as functional indicators. In short, if echolocation benefits blind people in real life it should be associated with a positive difference in any of these measures.

I used regression analyses to assess the role played by participants' use of echolocation while statistically controlling for their gender, age, level of visual function, general health, employment status, level of education, Braille skill, and use of other mobility means. I found that echolocation made a unique positive contribution to salary, and mobility in unfamiliar places, such that people who use echolocation had higher salary and higher mobility in unfamiliar places than people who did not use echolocation. The investigation was correlational in nature, and thus cannot be used to determine causality. In addition, caution must be exercised when generalizing results obtained with a small sample (sample size in current study was 37) to the population (Anderson and Vingrys, 2001). The data, however, are consistent with the idea that the use of echolocation has real life advantages for blind people.

## Materials and methods

All procedures were approved by the Applied Psychology Ethics Board at Durham University.

### Data collection

The survey was posted together with a letter of information on a publicly available internet page. Information about the survey was spread via word of mouth and by contacting organizations in contact with blind people. Specifically, an e-mail inquiry was sent that asked organizations if they were interested in spreading information about the survey for example by forwarding information about the survey to their members or by including information in a newsletter or online newsfeed. Together with the inquiry, information about the research, including ethical approval and data protection policies, and the address of the website that hosted the survey had been provided. Participants could access the survey and the letter of information by going to this website. The survey itself was a text file that participants downloaded. Blind participants can read web pages and electronic documents using screen-reader software, which converts written into spoken text. The majority of participants (36 out of 37) completed the survey by typing their answers into the text file and e-mailing it to the experimenter (LT). One participant contacted the experimenter (LT) by phone, and submitted answers over the phone. The first survey question solicited participants' informed consent. To assure confidentiality, participant's e-mail and e-mail address were deleted after their answers had been recorded. The survey was available from November 2011 until November 2012.

### Survey design

The first question of the survey solicited participant's informed consent. Questions 2–14 solicited information about the participant's gender, age, country of residence, cause of vision loss, age at which vision loss started, level of visual function, general health, employment status, salary, level of education, Braille skill, relationship status, use of mobility means, in that order. Question 8 used a single item response to solicit participant's opinion of their general health. Though single item responses are less detailed than longer measures of a person's health, they can be valid and reliable indicators (Bowling, 2005). Question 10 solicits information about participant's salary using salary categories rather than monetary value to bear on salary differences across countries. Questions 15 and 16 ask about the participant's mobility in familiar and unfamiliar environments, respectively. Previous studies have shown the usefulness of self-reported mobility measures in this form (Turano et al., 1999, 2002). The survey was available in English and German. Survey questions and the answer coding scheme for the English version are provided in the Appendix.

### Participants

37 people (18 female) responded to our survey. Respondents came from six different countries (UK: 13, USA: 4, Canada: 4, Germany: 14, Spain: 1, Australia: 1), mean age was 40.6 years (min = 18 ; max = 67 ; median = 37 ; *SD* = 14.4). For the majority of respondents vision loss was present or began at birth (*n* = 21, 56.8%), or it began before 16 years of age (*n* = 11, 29.7%) (see also Table 1). Twenty-two (59.2%) participants were totally blind. Reported cause of vision loss was heterogeneous, but the most commonly reported were Retinitis Pigmentosa (*n* = 6, 16.2%), Prematurity (*n* = 6), and Leber's Congenital Amaurosis (*n* = 4, 10.8%) (see also Table 2). The majority of participants (*n* = 30, 81.1%) considered themselves to be in generally good health. In terms of mobility means, by far the most commonly used mobility method used was the long cane (*n* = 34, 91.9%). This was followed by use of human guide (*n* = 16, 43.2%), echolocation (*n* = 10, 27%), GPS (*n* = 6, 16.2%), and guide-dog (*n* = 5, 13.5%) (for more details see Appendix, Question 14).

**Table 1:** **Summary of participants' responses to survey question 6** “**When did your vision loss start**.”

**Age in years when vision loss began**	**Frequency**	**Percent**
0	21	56.8
0.5	1	2.7
1.0	2	5.4
2.0	1	2.7
3.5	1	2.7
4.0	1	2.7
6.0	1	2.7
12.0	2	5.4
14.0	1	2.7
15.0	1	2.7
17.0	1	2.7
18.0	1	2.7
45.0	1	2.7
48.0	1	2.7
53.0	1	2.7
Total	37	100.0

*For the majority of participant vision loss was present or started at birth (56.8%)*.

**Table 2:** **Summary of participants' responses to survey question 5** “**What is the main cause of your vision loss**.”

**Cause of vision loss**	**Frequency**	**Percent**
Accident	2	5.4
Amaurosis	1	2.7
Blind born, glaucoma	1	2.7
Cone dystrophy	1	2.7
Genetic disorder, macular degeneration	1	2.7
Glaucoma, cataract	2	5.4
Glaucoma, macular degeneration	1	2.7
Leber's congenital amaurosis	4	10.8
Microphthalmia	1	2.7
Optic atrophy	1	2.7
Optic nerve atrophy	2	5.4
Optic nerve damage	1	2.7
Prematurity	4	10.8
Prematurity, glaucoma	1	2.7
Prematurity, retrolental fibroplasya	1	2.7
Retinal degeneration	1	2.7
Retinal detachment	2	5.4
Retinitis pigmentosa	3	8.1
Retinitis pigmentosa, alstrom syndrome	1	2.7
Retinitis pigmentosa, glaucoma	1	2.7
Retinitis pigmentosa, macular degeneration	1	2.7
Retinoblastoma	3	8.1
Virus during pregnancy	1	2.7
Total	37	100.0

Reported cause of vision loss was heterogeneous, but the most commonly reported were Retinitis Pigmentosa (n = 6, 16.2%), Prematurity (n = 6, 16.2%), and Leber's Congenital Amaurosis (n = 4, 10.8%).

### Data analysis

I investigated the following variables as markers of participants' functional abilities: salary, mobility in familiar places, mobility in unfamiliar places, and relationship status. To investigate the potential role that echolocation may play for each of these variables I conducted regression analyses for each of these measures separately.

For variables salary, mobility in familiar places and mobility in unfamiliar places I used stepwise linear regression with echolocation as predictor. In addition, I included participant's use of other mobility means, their sex, age, education level, employment status, Braille skill, general health, and their level of visual function as predictor variables. This way, the contribution through echolocation was evaluated while controlling for the contribution of the other variables. To follow up on linear regression results I used non-parametric tests. This was also done considering that the dependent variables were obtained using rating scales, and that therefore results obtained using parametric methods, such as linear regression, should be considered alongside non-parametric methods. As non-parametric measure of effect size I computed probability of superiority (PS) as suggested by Grissom and Kim (2012, pages 292–294), which estimates the probability that a score randomly drawn from one population will be greater than a score randomly drawn from another population.

For the variable relationship status I used binary logistic regression instead of linear regression to bear on the binary form of the response categories for those variables (relationship status: no relationship in past or present vs. relationship either in past or present). I used the forward likelihood ratio method for variable selection.

All statistical analyses were conducted using SPSS v20.0.

## Results

**Figure 1:**
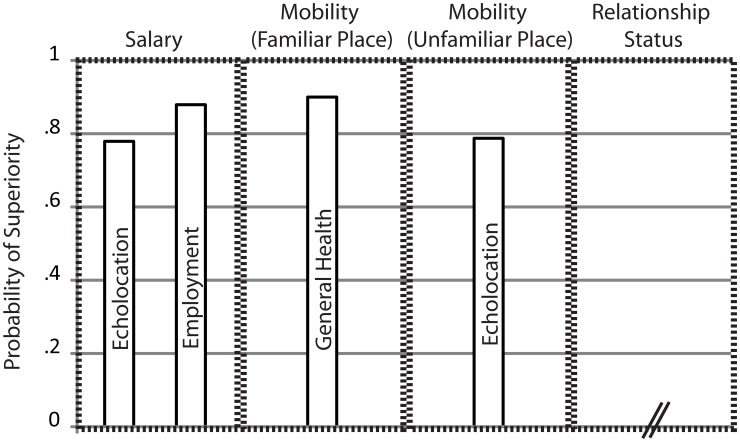
**Summary of Results.** Bars indicate non-parametric measures of effect-size, i.e., Probability of Superiority as suggested by Grissom and Kim (2012), for those predictors for which both linear regression coefficients and non-parametric tests were significant. Predictors are listed separately for variables “Salary,” “Mobility in Familiar Places,” “Mobility in Unfamiliar Places,” and “Relationship Status.” For the variable “Relationship Status” no predictor contributed significantly. For the other variables names of significant predictors are inscribed within each bar. Probability of Superiority estimates the probability that a score randomly drawn from one population will be greater than a score randomly drawn from another population. For example, Probability of Superiority of 0.78 for predictor “Echolocation” for variable “Salary” means that the probability that a randomly drawn salary score from an echolocating population will be greater than a randomly drawn salary score from a non-echolocating population is 0.78.

Figure 1 provides an overview of the results. Figure 1 shows effect sizes for those predictors for which both linear regression coefficients and non-parametric tests were significant.

### Salary

The regression showed that predictors echolocation [unstandardized coefficient *B* = 0.88; *t*_(31)_ = 2.42, *p* = 0.022] and employment status [unstandardized coefficient *B* = 1.65, *t*_(31)_ = 5.13, *p* < 0.001] contributed significantly to the overall model [*F*_(2, 31)_ = 20.121, *p* < 0.001, *R*^2^ = 0.562], whereas none of the other predictors were significant. Consistent with the regression results non-parametric tests for independent samples comparing salary between people who use echolocation and people who do not use echolocation was significant (Mann–Whitney *U* = 49, *p* = 0.01), as was the comparison between people who are employed and people who are not employed (Mann–Whitney *U* = 34.5, *p* < 0.001). Probability of superiority for echolocation (*PS*_Echolocation_) was 0.78. This means that the probability that a randomly drawn salary score from an echolocating population will be greater than a randomly drawn salary score from a non-echolocating population is 0.78. *PS*_Employment_ was 0.88. Weights for both predictors were positive, and *PS* for both predictors were larger than 0.5. Thus, the data suggest that people who are employed and/or people who use echolocation have higher salary. The finding that blind people who are employed have higher salary than blind people who are not employed makes intuitive sense and is consistent with previous data (e.g., Houtenville, 2003). The finding that the use of echolocation is associated with higher salary is novel.

### Mobility in familiar places

The regression showed that predictors general health [unstandardized coefficient *B* = 1.034, *t*_(33)_ = 3.392; *p* = 0.002], cane use [unstandardized coefficient *B* = 1.292; *t*_(33)_ = 3.25; *p* = 0.003] and education [unstandardized coefficient *B* = 0.241; *t*_(33)_ = 2.281; *p* = 0.029] contributed significantly to the overall model [*F*_(3, 33)_ = 13.816; *p* < 0.001; *R*^2^ = 0.557], whereas none of the other predictors was significant. Subsequent non-parametric tests comparing average mobility in familiar places between participants reporting good health and participants reporting not good health was significant (Mann Whitney *U* = 22; *p* < 0.001). *PS*_Health_ was 0.9. However, the comparison between cane users and cane non-users was not significant (Mann–Whitney *U* = 25.5, *p* = 0.116). Neither was the comparison across education levels (Kruskal–Wallis X^2^_(4)_ = 7.267; *p* = 0.122). The unstandardized coefficient for general health was positive, and *PS*_Health_ was larger than 0.5. Thus, in their entirety, the data suggest that good general health is associated with higher mobility in familiar places. Thus, blind people that report to be in good health also report to find it easier to move around in familiar environments than people that report to not be in good health. This finding is in agreement with previous research showing that better self-reported general health is related to better mobility (e.g., Harada et al., 1999).

### Mobility in unfamiliar places

The regression showed that predictors echolocation [unstandardized coefficient *B* = 0.755, *t*_(34)_ = 3.026; *p* = 0.005] and education [unstandardized coefficient *B* = 0.239; *t*_(34)_ = 2.43; *p* = 0.021] contributed significantly to the overall model [*F*_(2,34)_ = 9.326; *p* = 0.001; *R*^2^ = 0.354], whereas none of the other predictors were significant. Subsequent non-parametric tests comparing average mobility in unfamiliar places between participants reporting using echolocation and those not using echolocation were significant (Mann–Whitney *U* = 57; *p* = 0.003), and *PS*_Echolocation_ was 0.79. The comparison across various levels of reported education level was not significant [Kruskal–Wallis X^2^_(4)_ = 8.626; *p* = 0.071]. The unstandardized coefficient for echolocation was positive, and *PS*_Echolocation_ was larger than 0.5. Thus, in their entirety, the data suggest that the use of echolocation is associated with higher mobility in unfamiliar places. The finding that blind people who use echolocation find it easier to move around in novel places than blind people who do not use echolocation is novel.

### Relationship status

None of the predictors contributed significantly to the overall model.

## Discussion

Based on the hypothesis that blind people use echolocation because it offers functional benefits I would expect that there should be measurable functional benefits for blind people who echolocate, as compared to blind people who do not echolocate. As laid out in the introduction, consistent with this hypothesis previous research under controlled experimental conditions has shown that echolocation improves blind people's spatial sensing abilities [for reviews see Stoffregen and Pittenger (1995) and Schenkman and Nilsson (2010)]. The current study investigated if there is also evidence for functional benefits of echolocation in real life. In an opportunity sample of 37 participants I found that echolocation was associated with higher salary and mobility in unfamiliar places. This finding is consistent with the idea that echolocation may indeed have functional advantages for blind people in real-life.

The sample size was relatively small (37 participants total, 10 echolocators). This has implications both for statistical analyses and sampling veracity.

With regard to statistical analyses low sample sizes may lead to low statistical power, as well as to problems using parametric statistical procedures, i.e., the regression approach. For the current study the use of parametric procedures can also be considered problematic because some dependent measures were obtained via rating scales. One point to consider in this context is that regression results were always followed up using non-parametric tests, confirming that the results hold also when using distribution free testing methods. In addition, despite low sample size reliable effects for echolocation were found for mobility in new places and salary, so lack of statistical power *per se* is not an issue.

With regard to sampling veracity the small sample size implies that one must be cautious when generalizing the current results to the population (Anderson and Vingrys, 2001). The survey was available for 12 months, and participation was solicited through word-of-mouth and advertising through various organizations in contact with blind people. Despite these efforts we received only 37 responses, exemplifying previously reported problems with the soliciting of participation of blind participants for survey research. For example, Turano et al. (1999) sent out 299 questionnaires and after three attempts of mailing the surveys and telephoning each participant to take part via audio response, stressing the difficulty of gathering a large enough population sample, only 145 (under 50%) were returned. Nzegwu and Dooley (2008) managed to collect only 94 responses despite sending letters to 5000 parents and 1500 children and data collection spanning over a year. In sum, the size of our sample necessitates that caution is needed when generalizing the current findings to the population. Similarly, our sample was comparably young and comprised a large number of people who had lost sight early in life. In the overall demographic of blind people, there is a large number of people who lose vision in old age. In fact, in the year 2002 82% of the 314 million visually impaired adults recorded by the World Health Organization were 50 years and older at time of onset of blindness (Resnikoff et al., 2004). It follows that people who lose sight in old age are under-represented in our sample. Older people tend to make less use of the Internet (Kaye, 2000). Thus, the most likely reason for the low number of older people in our sample is that the survey was posted online. Future research is needed to determine if similar results will be obtained in a sample that includes larger numbers of people who lost sight in old age.

Another point to consider is that our study is correlational in nature. As such, we cannot determine if the use of echolocation causes better salary and mobility in unfamiliar environments, or if people who have better mobility in unfamiliar environments and higher salary also echolocate. With regard to mobility in unfamiliar environments, however, previous laboratory research supports the idea that echolocation may actually cause improvements. Specifically, previous research shows that echolocation improves spatial sensing. Blind people can use echolocation for example to detect a 3-degree change in the horizontal position of an object placed 1.5 m away (Thaler et al., 2011) or a gap as small as 0.02 m between two objects placed 1 m away (Teng et al., 2012), or a 4″ displacement in depth at a distance of 90 cm (Kellogg, 1962). This would suggest that the use of echolocation would also lead to improved mobility in unfamiliar environments, where mobility cannot rely on memory, but requires the exploration of a novel spatial layout. With regard to salary, there is no previous laboratory research, but it would seem improbable that the use of echolocation *per se* would lead to an increase in salary. However, it is possible that for example the increased mobility in unfamiliar places as mediated though echolocation may have a positive impact on blind people's professional autonomy and in this way also on their salary.

The majority of our participants use the long cane, and all of our participants who echolocate, also use the long cane. This suggests that the benefit of echolocation we found might be conditional upon the long cane being used as well. It also suggests that echolocation and long cane may have complementary functions. For example, it is possible that the cane is more beneficial to sense the layout of the ground surface, which might be challenging to sense through echolocation because the overall sound reflection of the ground surface may mask more subtle changes in layout, such as a rising curb or a pothole. In contrast, echolocation may be more useful to sense surfaces elevated off the ground around head level, where the cane is inconvenient to apply, and/or may pose risks to other people in the environment.

The design of our survey was deliberate. As such, no investigation was made into matters such as questionnaire validity or reliability, or to what degree the phrasing or ordering of questions may have influenced participants' answers. With regard to the solicitation of the use of mobility means, the different techniques were simply listed and for example no particular definition of echolocation was used (compare Question 14 in the Appendix). One might argue, therefore that only respondents familiar with this technique would respond positively, and that knowledge of echolocation might perhaps be related to educational level. Our statistical analyses, however, that controlled for educational level, suggest that differences in educational level cannot explain our findings. Finally, most of the questions solicited demographic information, or they were chosen based on the previous literature. In sum, we think that it is unlikely that the results are due to methodological artifacts related to the design of the questionnaire.

As mentioned in the introduction, previous research suggests that up to 30% of blind people may echolocate. Consistent with this, 10 out of the 37 people in the sample reported to use echolocation. Since previous laboratory studies as well as our current data suggest that the use of echolocation may lead to functional benefits for blind people, the question arises, why not more blind people echolocate. One possible explanation is lack of knowledge.

An early description of a blind person avoiding obstacles was given by Diderot in 1749. Initially the mechanisms underlying this “obstacle sense” were unclear, and it was thought that it might be an ability of only a few gifted people. In the 1940's/1950's, however, it became clear that echolocation was auditory in nature, and that anybody with normal hearing can learn it (Supa et al., 1944; Cotzin and Dallenbach, 1950; Worchel and Mauney, 1951; Ammons et al., 1953; Teng and Whitney, 2011). Thus, lack of knowledge might not be the (sole) reason for echolocation not being used more by blind people. Another possibility for the limited popularity of echolocation might be concerns about social stigma. Specifically, echolocation requires people to generate sonar emissions, such as finger snaps, shuffling with their feet, clicking with their tongue, humming, repetitive speaking, etc., and blind people may be reluctant to produce sonar emissions out of concern to appear “odd.” In addition, behaviors in blind people that “appear to have no goal directed purpose” and that appear to be out of the norm are considered maladaptive mannerisms, sometimes also referred to as “Blindisms” and they are discouraged from an early age (Eichel, 1978; Molloy and Rowe, 2011). There is the possibility that spontaneously generated sonar emissions might be considered maladaptive mannerisms and therefore be discouraged, and this will affect the degree to which blind people will make use of echolocation.

In summary, our data are consistent with the idea that echolocation offers functional benefits for blind people in real life. This finding, together with previous laboratory research, provides converging evidence for the idea that echolocation may play a role in peoples' successful adaptation to vision loss.

### Conflict of interest statement

The author declares that the research was conducted in the absence of any commercial or financial relationships that could be construed as a potential conflict of interest.

## Appendix

This Appendix contains survey questions and response options. The response coding scheme as well as the frequency of responses in the different response categories are shown below each question.

1. Please tell us if you have read the letter of information and consent to participate in this survey
  [Yes:1(37), No: 0(0)]
2. What is your gender
  [male: 0(19), female: 1(18)]
3. What is your date of birth
  (mm/yyyy) (mean = 40.6 years, min = 18; max = 67; median = 37; *SD* = 14.4)
4. Where do you live currently
  (country) (UK: 13, USA: 4, Canada: 4, Germany: 14, Spain: 1, Australia: 1)
5. What is the main cause of your vision loss
  (free text answer) see Table 2
6. When did your vision loss start
  (mm/yyyy) see Table 1
7. Please describe the level of your visual function
  [total blindness: 0(22), partial vision: 1(15)]
8. Do you consider yourself to be in generally good health?
  [No: 0(7), Yes: 1(30)]
9. What is your current employment status
  [going to school: 0(2), going to university: 0(11), unemployed: 0(4), employed full time: 1(10), employed part time: 1(5), self employed: 1(1), retired: 0(4)]
10. How would you describe your salary
  [none: 0(4), benefits: 1(12), minimum wage: 2(2), above minimum wage: 3(11), high income: 4(5), missing: (3)]
11. How would you describe your level of education
  [none: 0(0), primary/middle school: 1(4), secondary/high school: 2(13), Bachelors degree: 3(7), Masters degree: 4(11), Doctoral degree: 5(2)]
12. What is your skill in reading Braille
  [none: 0(11), little: 1(2), fluent: 2(24)]
13. Please indicate your current relationship status
  [single without previous relationship: 0(7), single with previous relationship: 1(11), currently in a relationship: 1(18), missing: (1)]
14. Which mobility means do you use
  [partial vision, Yes: 1(4), No: 0(33)]
  [long cane, Yes: 1(34), No: 0(3)]
  [guide dog, Yes: 1(5), No: 0(32)]
  [human guide, Yes: 1(16), No: 0(21)]
  [echolocation, Yes: 1(10), No: 0(27)]
  [auditory sensory substitution device, Yes: 1(2), No: 0(35)]
  [tactile sensory substitution device, Yes: 1(0), No: 0(37)]
  [GPS, Yes: 1(6), No: 0(31)]
  [Other, Yes: 1(2), No: 0(35), monocular, compass]
15. When you are on your own in a familiar place, how difficult do you find it to find your way around
  [very difficult: 0(3), somewhat difficult: 1(3), somewhat easy: 2(11), very easy: 3(20)]
16. When you are on your own in a new place, how difficult do you find it to find your way around
  [very difficult: 0(6), somewhat difficult: 1(22), somewhat easy: 2(6), very easy: 3(3)]
